# A naturally occurring InDel variation in *BraA.FLC.b* (*BrFLC2*) associated with flowering time variation in *Brassica rapa*

**DOI:** 10.1186/1471-2229-12-151

**Published:** 2012-08-28

**Authors:** Jian Wu, Keyun Wei, Feng Cheng, Shikai Li, Qian Wang, Jianjun Zhao, Guusje Bonnema, Xiaowu Wang

**Affiliations:** 1 Institute of Vegetables and Flowers, Chinese Academy of Agricultural Sciences, Beijing, 100081, China; 2 Institute of Horticultural Science, Yunnan Academy of Agricultural Sciences, Kunming, 650205, China; 3 Laboratory of Plant Breeding, Wageningen University, Droevendaalsesteeg 1, 6708 PB, Wageningen, The Netherlands

## Abstract

**Background:**

Flowering time is an important trait in *Brassica rapa* crops. FLOWERING LOCUS C (FLC) is a MADS-box transcription factor that acts as a potent repressor of flowering. Expression of FLC is silenced when plants are exposed to low temperature, which activates flowering. There are four copies of *FLC* in *B. rapa*. Analyses of different segregating populations have suggested that *BraA.FLC.a* (*BrFLC1*) and *BraA.FLC.b* (*BrFLC2*) play major roles in controlling flowering time in *B. rapa*.

**Results:**

We analyzed the *BrFLC2* sequence in nine *B. rapa* accessions, and identified a 57-bp insertion/deletion (InDel) across exon 4 and intron 4 resulting in a non-functional allele. In total, three types of transcripts were identified for this mutated *BrFLC2* allele. The InDel was used to develop a PCR-based marker, which was used to screen a collection of 159 *B. rapa* accessions. The deletion genotype was present only in oil-type *B. rapa*, including ssp. *oleifera* and ssp. *tricolaris*, and not in other subspecies. The deletion genotype was significantly correlated with variation in flowering time. In contrast, the reported splicing site variation in *BrFLC1*, which also leads to a non-functional locus, was detected but not correlated with variation in flowering time in oil-type *B. rapa*, although it was correlated with variation in flowering time in vegetable-type *B. rapa.*

**Conclusions:**

Our results suggest that the naturally occurring deletion mutation across exon 4 and intron 4 in *BrFLC2* gene contributes greatly to variation in flowering time in oil-type *B. rapa*. The observed different relationship between *BrFLC1* or *BrFLC2* and flowering time variation indicates that the control of flowering time has evolved separately between oil-type and vegetable-type *B. rapa* groups.

## Background

*Brassica rapa* is a genus comprising a variety of vegetables such as Chinese cabbage (ssp. *pekinensis*), pak choi (ssp. *chinensis*), and turnip (ssp. *rapa*) as well as oil crops including turnip rape (ssp. *oleifera*) and sarson (ssp. *tricolaris*). Flowering time is an important trait in Brassica vegetables because early flowering often leads to low yield and low quality. It is also important for oilseed rape varieties as they are divided into “winter” and “spring” types according to their different flowering times and responsiveness to vernalization. Winter types must be exposed to cold to transition from the vegetative growth stage to the reproductive stage, while this is not necessary for the spring types, which are generally grown in shorter-season areas.

In *Arabidopsis*, studies of natural variation demonstrated that the vernalization requirement is largely conferred by two dominant genes, FRI and FLC [1-3]. FRI acts upstream of FLC to positively regulate FLC expression [4]. *FLC* encodes a MADS-box transcription factor that functions as a repressor of flowering by inhibiting downstream floral integrator genes [5-9]. Vernalization represses the expression of *FLC* and induces flowering. The promoter and first exon of *FLC* are sufficient to initiate the repression of *FLC* during vernalization, while the maintenance of repression requires additional regions of the gene body [10].

There are four copies of *FLC* in *B. rapa*[11-13]. They are located on chromosomes A10 (*BraA.FLC.a*, named *BrFLC1*), A02 (*BraA.FLC.b*, named *BrFLC2*), and A03 (*BraA.FLC.c*, named *BrFLC3* and *BraA.FLC.d*, named *BrFLC5*) owing to polyploidy evolution [13,14]. Colinearity analysis indicated that *BrFLC1*, *BrFLC2*, and *BrFLC3* are located in three R blocks (Xiaowu Wang *et al*. unpublished data), which is consistent with the three *FLC* copies that would be expected after a triplication event [12-15]. *BrFLC5* is located between blocks I and J [16]. Multiple gene copies are thought to be responsible for dose-regulated expression, and the mechanism appears to affect variations in flowering time in *Brassica* crops [17]. These replicated genes may have additive effects. Depending on the specific cross studied, different alleles of the various *FLC* paralogs may exert different effects on flowering time. In a backcross population derived from two recombinant inbred lines, created from a cross between Per and R500, a quantitative trait locus(QTL) co-located with *BrFLC1* explained more of the flowering time variation than *BrFLC2*[12]. It was also reported that a naturally occurring splicing variation in *BrFLC1* was associated with variation in flowering time in *B. rapa*, and this locus contributed most of its effect to late flowering [18]. However, Zhao *et al.*[19] studied a doubled haploid (DH) population derived from a cross between pak choi and yellow sarson, and reported *BrFLC2* as a candidate gene for a major QTL for flowering time and the vernalization response in *B. rapa*. The decreased transcript level of *BrFLC2* upon cold treatment provided further evidence for this hypothesis.

Since there are apparent contradictions in the proposed roles of *BrFLC1* and *BrFLC2*, we analyzed sequence variation of *BrFLC1* and *BrFLC2* in a large collection of *B. rapa* accessions, and the relationship between sequence variations and flowering time. Our results indicate that among the various *B. rapa* crop types, there are different genetic controls of flowering time.

## Results

### Flowering time variation

We determined flowering time for the germplasm collection of 159 *B. rapa* accessions in two separate experiments; one in an open field in Kunming, South China, and one in a heated greenhouse in Beijing, North China. The data from the two experiments were significantly correlated with each other (R^2^ = 0.68, P ≤ 0.01). The flowering time varied from 52 to 155 days from sowing to the opening of the first flower (days to flowering, DTF) in the open field experiment, and 42 to 150 DTF in the greenhouse experiment (Table 1, Additional file 1). Flowering time varied more within turnip rape than within the other subspecies, with a variation range of 87 days in the open field to 104 days in the greenhouse. The three yellow sarson accessions showed the narrowest variation (5 days in the open field, 3 days in the greenhouse). The pak choi accessions showed the greatest difference in flowering time between the two experiments, indicating that day length likely affected the timing of flowering more than vernalization, since the average day length was more than 200 h per month during December to April in Kunming, compared with 170–190 h per month in Beijing. In the open field experiment, among the vegetable-type *B. rapa* accessions, Caixin and Zicaitai flowered earlier, with an average flowering time of 82 DTF and 90 DTF, respectively, while both Chinese cabbage and pak choi flowered at 124 DTF on average. Turnips flowered latest, 142 DTF on average, followed by Wutacai (average, 133 DTF). The results from the greenhouse experiments were largely consistent with those obtained from the open field experiments (Table 1, Additional file 1).

**Table 1:** **Flowering time variation among the germplasm collection of*B. rapa***

**Cultivar Group**	**Subspecies**	**No. of Acc.**	**Open field**		**Greenhouse**	
			**Ave. DTF**	**Var. range (d)**	**Ave. DTF**	**Var. range (d)**
Turnip	ssp. *rapa*	23	142	113-155	143	108-150
Chinese cabbage	ssp. *pekinensis*	5	124	99-136	118	69-138
Pak choi	ssp. *chinensis*	29	124	103-154	121	56-150
Caixin	ssp. *parachinensis*	11	82	52-115	70	50-108
Broccoletto	ssp. *broccoletto*	4	105	74-152	108	80-150
Zicaitai	ssp. *chinensis* var. *purpurea*	7	90	52-117	97	55-126
Mizuna	ssp. *nipposinica*	1	142	-	136	-
Neep greens	ssp. *perviridis*	2	123	115-130	143	135-150
Wutacai	ssp. *narinosa*	3	133	127-139	133	121-142
Turnip rape	ssp. *oleifera*	71	86	67-154	85	46-150
Yellow sarson	ssp. *tricolaris*	3	77	74-79	55	53-56

Acc., Accessions; Ave. DTF, Average days to flowering; Var., variation.

### Nucleotide polymorphisms at the *BrFLC2* gene

**Figure 1:**
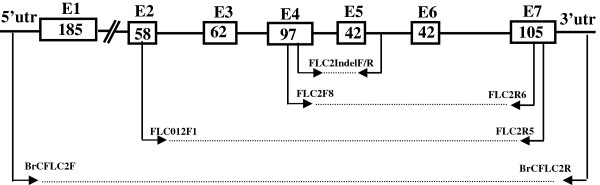
***FLC2*primers used in this study.** (**a**) Genomic structure of *A. thaliana FLC* gene (from [12]). E, exon; exon sizes shown by white boxes. (**b**) FLC2IndelF/R: *FLC2*-specific primers used to screen for 57-bp InDel polymorphisms across exon 4 and intron 4. (**c**) FLC2F8/FLC2R6: *FLC2*-specific primers in exon 4 and exon 7. (**d**) FLC012F1/FLC5: *FLC2*-specific primers in exon 2 and exon 7. (**e**) BrCFLC2F/R: *FLC2*-specific primers in 5′UTR and 3′UTR.

**Figure 2:**
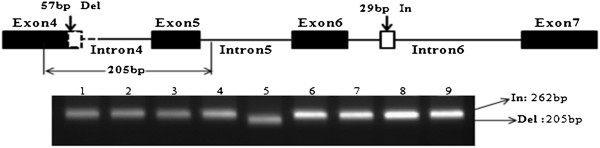
**Schematic model of development of InDel marker used to detect 57-bp deletion mutation across exon 4 and intron 4 of*BrFLC2*.** (**a**) Insertion and deletion identified in *B. rapa* ssp. *olerferious*. Del, deletion; In, insertion. Amplified region used to detect deletion mutation covered part of exon 4, intron 4, and part of exon 5. (**b**) Amplified fragments. In and Del represent insertion allele and deletion allele, respectively, followed by fragment size (in base pairs). Nine accessions (1–9) are listed in same order as in Table 2 .

Nine accessions were selected to sequence *BrFLC2* (Table 2). These nine accessions represented seven *B. rapa* subspecies, and showed a wide range of variation in flowering times. For six of the nine accessions, flowering time varied from 38 to 66 DTF in the greenhouse in 2009. The other three accessions (L203, CGN06818, CGN15202) had not flowered by 90 days after sowing. To investigate the allelic variation in *BrFLC2*, a 1400-bp fragment was amplified from *B. rapa* genomic DNA with the primer combination of FLC2F8 in exon 4 and FLC2R6 in exon 7 (Figures 1). Sequencing of the amplified fragments from the nine selected accessions revealed that the amplified region had multiple sequence variations among the nine accessions. All of these polymorphisms were single nucleotide substitutions, with two additional insertion/deletions (InDel) in line L143, a yellow sarson accession. One was a deletion of 57 bp, started at 1851 bp of *BrFLC2* (Bra028599, http://brassicadb.org/brad/) and ended at 1914 bp, across exon 4 and intron 4. The deletion was interrupted by 5 nucleotides (TAAAT) that could not be mapped to a certain position of the reference sequence (Figures 2, Additional file 2). The other one was an insertion of 29 bp at the position of 2430 bp located in intron 6 of *BrFLC2*. All the single nucleotide substitutions were synonymous. An InDel marker designated as *BrFLC2*InDel was developed to distinguish the 57 bp deletion across exon 4 and intron 4 and validated in the nine sequenced accessions (Figures 2).

**Table 2:** **Nine *B. rapa* accessions used to sequence *BrFLC2* in this study**

**Accessions**	**Subspecies**	**Cultivar group**	**Flowering behavior**
HN54	ssp. *pekinensis*	Chinese cabbage	annual, early
L144	ssp. *oleifera*^a^	Rapid cycling	annual, very early
L77	ssp. *pekinensis*	Chinese cabbage	annual, early
Z16	ssp. *pekinensis*	Chinese cabbage	annual, early
L143	ssp. *tricolaris*	Yellow sarson	annual, early
L203	ssp. *nipposinica*	Mizuna	biannual
CGN15202	ssp. *perviridis*	Neep greens	biannual
CGN06818	ssp. *rapa*	Turnip	biannual
L58	ssp. *parachinensis*	Caixin	annual, early

^a^[20].

### Relationship between flowering time and nucleotide polymorphisms in *BrFLC2*

The *BrFLC2* InDel was screened over the germplasm collection consisting of 159 accessions. The deletion allele was absent from all of the vegetable-type subspecies, but it was present as a homozygous allele in nine out of 71 *B. rapa* ssp. *oleifera* accessions and in all three of the ssp. *tricolaris* accessions, and as a heterozygous allele in 13 *B. rapa* ssp. *oleifera* accessions (Table 3, Additional file 1). The correlation analysis showed that the InDel polymorphism was significantly associated with variation in flowering time among the oil-type *B. rapa* accessions (Table 3). In all cases, accessions with the deletion allele flowered early, with an average flowering time measured as days to flowering (DTF) of 72 in the open field experiment and 62 DTF in the greenhouse experiment. In contrast, the accessions without this deletion (i.e. those with the functional allele) flowered significantly later (P ≤ 0.001) with an average DTF of 90 in the open field experiment and 93 in the greenhouse experiment. The flowering time of the accessions with the heterozygous *BrFLC2* locus were similar to those of accessions with the homozygously mutated locus, indicating that early flowering was dominant over late flowering.

**Table 3:** **Flowering time variation among germplasm collection of *B. rapa***

**Genotype**	**Vegetables**			Oil type*		
	**No. of lines**	**DTF Open field**	**DTF Greenhouse**	**No. of lines**	**DTF Open Field**	**DTF Greenhouse**
*BrFLC1*						
A	45	108 ± 25b	105 ± 45 b	38	85 ± 12a	84 ± 25 a
G	37	132 ± 20a	137 ± 22 a	11	92 ± 25a	83 ± 33 a
H	3	112 ± 12b	115 ± 35 b	25	83 ± 12a	83 ± 18 a
*BrFLC2*						
insertion	85	119 ± 26	118 ± 30	49	90 ± 15a	93 ± 23 a
deletion				12	72 ± 4b	62 ± 18 b
H				13	78 ± 8b	70 ± 12 b

DTF (days to flowering) data are presented as means ± SE. Significant differences (P ≤ 0.001) between lines are indicated by different letters.

*Includes ssp. *oleifera* and ssp. *tricolaris*.

We further analyzed the 159 accessions using the previously reported *BrFLC1MvaI* CAPS marker, which can distinguish A/G alleles located at the splicing site of exon 4 and intron 4 of *BrFLC1*. The A allele results in alternative splicing, which is correlated with early flowering in *B. rapa*[18]. *BrFLC1* allelic variation was observed in both vegetable-types and oil-types, showing A, G, and heterozygous alleles (Table 3). The flowering time in vegetable-type *B. rapa* was significantly correlated with the A/G genotype; accessions with the A genotype had an average flowering time of 108 DTF in the open field experiment and 105 DTF in the greenhouse experiment. Accessions with G genotype flowered at 132 DTF in the open field experiment and 137 DTF in the greenhouse experiment. However, there was no significant relationship between the *BrFLC1* A/G genotype and variations in flowering time among oil-type *B. rapa* accessions. The mean DTF of the 38 oil-type accessions with the A allele was 85 in the open field experiment and 84 in the greenhouse experiment. In contrast, the average flowering time of 11 accessions with the G allele was 92 DTF in the open field experiment and 83 DTF in the greenhouse experiment. Similar to *BrFLC2*, early flowering controlled by *BrFLC1* was dominant over late flowering (Table 3).

The association between flowering time and the *BrFLC2* InDel alleles was also analyzed for DH progenies from a BC_2_ population, using a Chinese cabbage accession Z16 as the recurrent parent and yellow sarson accession L143 as the donor. Both parents had “A” alleles for *BrFLC1*, while Z16 had a *BrFLC2* allele without deletions that was possibly functional, and L143 had the deletion allele of *BrFLC2*. Of the 120 screened DH lines, 3 had the deletion allele. We investigated the flowering phenotype of these three lines, five randomly selected lines with the functional allele, and the two parental lines. The lines with the deletion allele showed significantly shorter flowering times than those of lines with the functional allele (Table 4). The flowering times of DH lines with the functional *BrFLC2* allele ranged from 83 DTF to 92 DTF, while those of lines with the mutated deletion allele ranged from 70 DTF to 80 DTF.

**Table 4:** **Flowering time in BC**_**2**_**DH progenies with different *BrFLC2* alleles**

**Line**	***BrFLC2*allele**	**DTF**
Z16	In	83 ± 1.64cde
L143	Del	78 ± 3.13ef
WJ10Q011	Del	80 ± 2.41def
WJ10Q012	Del	70 ± 2.00 g
WJ10Q013	Del	76 ± 1.52 fg
WJ10Q014	In	85 ± 1.00bcd
WJ10Q015	In	89 ± 0.00abc
WJ10Q016	In	91 ± 2.17ab
WJ10Q017	In	92 ± 2.19a
WJ10Q018	In	83 ± 1.37cde

DTF (days to flowering) data are presented as means ± SE. Significant differences (P ≤ 0.001) between lines are indicated by different letters.

### Alternative splicing of *BrFLC2*

**Figure 3:**
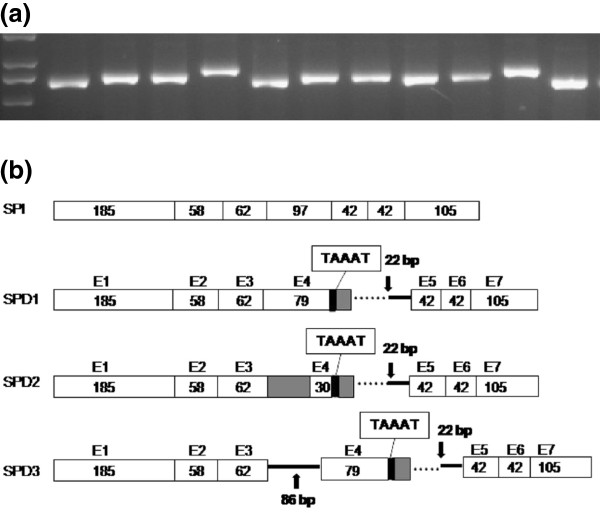
**Sequence variation affects RNA splicing.** (**a**) PCR fragments amplified from 11 *E. coli* clones transformed with *BrFLC2* RT-PCR products from yellow sarson accession L143. (**b**) Three alternative splicing patterns identified in accession L143. SPI: constitutive splicing pattern [11]; SPD1–3: alternative splicing patterns. White boxes indicate exons; shaded box, missing parts of exon 4; dotted lines, intron parts missing from transcripts; solid lines, retained parts of introns; black bars, the 5 bp insertion (TAAAT).

To identify any alternative splicing of the mutated *BrFLC2* allele, RT-PCR was conducted with *BrFLC2*-specific primers (BrCFLC2F and BrCFLC2R) using the yellow sarson accession L143, which had a homozygous deletion allele. Three fragments of different lengths were amplified from the cDNA of L143 (Figures 3). Sequencing of these fragments revealed three alternative splicing patterns. The 5 nucleotides (TAAAT) which interrupted the 57 bp deletion were found in transcripts from all three splicing patterns. The three patterns were as follows: SPD1) 600-bp transcript—18 bp skipped at the end of exon 4 following the (TAAAT) insertion and 22 bp retained at the end of intron 4; SPD2) 551-bp transcript—49 bp skipped at the beginning and 18 bp skipped at the end of exon 4, the retained 30 bp of exon 4 followed by the (TAAAT) insertion and 22 bp retained at the end of intron 4; and SPD3) 686-bp transcript—same as SPD1 besides intron 3 retained. Of the 36 sequenced cDNA clones, 28 showed SPD1, six showed SPD2, and two showed SPD3. This indicated that the SPD1 was the major splicing pattern for the deletion allele of *BrFLC2*, while SPD2 and SPD3 were derived from SPD1.

## Discussion

An InDel polymorphism across exon 4 and intron 4 of *BrFLC2* was discovered in a subset of oil-type *B. rapa* accessions, including ssp. *oleifera* and ssp. *tricolaris*. In this study, we investigated its relationship with flowering time both in a collection of *B. rapa* natural accessions and in a BC_2_DH population.

Plants need to sense their environment and initiate flowering at the appropriate time to ensure successful fertilization and production of abundant seeds. There is considerable variation in the flowering time among, but also within, natural populations, as we observed in the present study of a *B. rapa* germplasm collection. In *A. thaliana*, the *FRI* gene was shown to be a major determinant of flowering time variation in the natural population through its effects on the expression of *FLC*[21,22]. An InDel variation in the *COL1* gene was reported to be correlated with variation in flowering time in *B. nigra*[23]. In *A. thaliana*, *FLC* encodes a MADS-box transcription factor that acts as a dose-dependent flowering repressor [9,22]. Four copies of the *FLC* gene in *B. rapa* increase the potential variation in flowering time [17]. In our previous study, we identified a splicing site polymorphism Pi6 + 1(G/A) in *BrFLC1* that was significantly associated with the naturally occurring variation in flowering time in *B. rapa*[18]. In that study, we examined 96 lines, six of which were oil-types. In contrast, half of the lines examined in the present study were oil-types. Because there were so few oil-types in our previous study, we did not identify that oil-type and vegetable-type *B. rapa* showed different relationships between alleles of *BrFLC1* and flowering time variation. In the present study, we could not detect any effect of allelic variation in *BrFLC1* Pi6 + 1(G/A) on the variation in flowering time for oil-type *B. rapa*, including ssp. *oleifera* and ssp. *tricolaris*. However, we detected an InDel polymorphism across exon 4 and intron 4 of *BrFLC2* among the accessions of ssp. *oleifera* and ssp. *tricolaris*. This sequence polymorphism was not detected in any of the other vegetable-type *B. rapa* subspecies. Furthermore, this allelic variation was strongly associated with variations in flowering time in oil-type *B. rapa*. Zhao *et al*. [19] suggested that *BrFLC2* was a major determinant of flowering time variation in *B. rapa.* However, there were no firm conclusions from several studies on *BrFLC1*[12,18] and *BrFLC2*[19]. Since *BrFLC1* and *BrFLC*2 have specific roles in controlling flowering time in different *B. rapa* groups, we deduced that there was an independent evolution of the control of flowering time, at least the control of the vernalization pathway, between oil-type *B. rapa* including ssp. *oleifera* and ssp. *tricolaris* and the other vegetable-type and turnip *B. rapa* subspecies. The oil-type *B. rapa* formed an evolutionary branch that was independent of other *B. rapa* species in an analysis of molecular phylogeny based on whole genome re-sequencing data generated from 108 accessions (Dr. Xiaowu Wang, unpublished data). This indicates that the evolutionary history of oil-type *B. rapa* is isolated from that of the vegetable-type subspecies. The fact that the deletion mutation of *BrFLC2* was absent from vegetable-type *B. rapa* indicates that this mutation may have arisen after the division of oil-type from vegetable-type *B. rapa*, while the splicing site mutation of *BrFLC1* may have arisen before this division and been maintained during their respective evolutions.

Relationships between naturally occurring alternative splicing variants and flowering time variation have been reported for the *FLC* gene in *A. thaliana*[24] and *Capsella bursa-pastoris*[25], and for *BrFLC1* in *B. rapa*[18]. Alternative splicing variants were also reported for *BrFLC5* in a biennial oilseed cultivar, although their relationship with flowering time was not addressed [12]. In the present study, we detected three alternative splicing patterns for B*rFLC2* in the yellow sarson accession L143, which has a homozygous deletion allele of *BrFLC2*. All three alternative splicing variations led to the insertion of premature stop codons in the transcripts. The alternative splicing pattern iii of *BrFLC2* has been reported by Zhao et al. [19] using DH lines derived from a cross between the same yellow sarson accession and a pak choi accession, and was deduced to be a regulatory mechanism for the differential expression of *BrFLC2* in response to vernalization. In the present study, the transcripts from splicing pattern iii were the minor fraction of transcripts from the deletion allele of *BrFLC2.* This could be due to the different cultivation conditions in the two studies, as the plants were not cold-treated in this study. A possible reason for the differential expression in response to cold treatment might be that alternative splicing transcripts were eliminated by the mRNA surveillance system. Eukaryotes have an mRNA surveillance system to eliminate the transcripts that are deliberately spliced to contain premature stop codons as a part of their intricate autoregulatory system [26].

*B. rapa* is a mesohexaploid that has undergone whole genome triplication after divergence from a common ancestor of *A. thaliana*. During the diploidization process afterwards, which involved considerable gene loss, some gene family showed preferential retention such as circadian clock genes [27], and also many of flowering time genes*.* It has been speculated that polyploidy and lost of the duplicated genes may have contributed to the evolution of variations in flowering time, a key component of morphological diversity [17]. After the hexaploid process, the three sub-genomes of the ancestor were partitioned into LF, MF1, and MF2 [13]. *BrFLC1* is located in LF, *BrFLC2* in MF2, and *BrFLC3* in MF1 [13], while *BrFLC5* is located in the homologous region generated from an α-duplication event that occurred before the diversification of *Arabidopsis-Brassica*[14]. The fact that a non-functional *BrFLC1* mutation introduces early flowering time variation in vegetable-type *B. rapa*, while the non-functional *BrFLC2* introduces early flowering time variation in oil-type *B. rapa*, indicates that these two loci of *FLC* in *B. rapa* play different roles in different groups. It has been proposed that non-functionalization of duplicate genes could provide an important source of phenotypic variation [25]. We have shown that the deletion in *BrFLC2* also promoted flowering in a genetic background of Chinese cabbage line Z16. However, it remains unknown why different alleles of *BrFLC1* show no difference in flowering time in oil-type *B. rapa* accessions. We need to sequence all of the *BrFLC1* alleles in these accessions to determine whether they contain additional mutations. Genetic redundancy provides flexibility for plants growing in changeable environments. It is also possible that the other two homologs of *FLC* might function to compensate for the loss of function of *BrFLC1* or *BrFLC2*. We sequenced *BrFLC3* or *BrFLC5* using primers designed from sequences in exon 4 and exon 7, respectively, and we did not identify any functional sequence variation for the nine accessions (unpublished data). However, we can not exclude the possibility that there are sequence variations located in other regions that might affect their functions. Further research on *BrFLC3* in sub-genome MF1 and *BrFLC5* as a relic of the α-duplication event and their influence on flowering is underway. We anticipate that they may have differentiated from, and are functionally different from, *BrFLC1* and *BrFLC2*.

## Conclusions

Our results suggest that the naturally occurring deletion mutation across exon 4 and intron 4 in *BrFLC2* contributes greatly to the variation in flowering time in oil-type *B. rapa*. The different relationships between *BrFLC1* or *BrFLC2* and the variation in flowering time of vegetable-type and oil-type *B. rapa* indicate that control of flowering time undergone separate evolution between these two groups.

## Methods

### Plant materials and flowering time evaluation

To characterize the natural variation of flowering time in *B. rapa*, we measured 159 accessions belonging to 11 cultivar groups (Table 1, Additional file 1). We investigated flowering time for this germplasm collection under open field conditions in Kunming, Yunnan Province, southern China, from 12 October 2009 to 1 April 2010, as well as in a greenhouse in Beijing, northern China, from 23 October 2010 to 20 March 2011. The lowest mean daily temperature in Kunming is in December and January (average, 8.2°C). The day length varied between 142.7 h in October and 244.8 h in April. The day length in Beijing is 2,562–2,744 h per year on average. The shortest days are in winter (581.6 h in total during December to February), and the longest days are in spring (778.8 h in total from March to May). The temperature in the greenhouse varied between 15 to 25°C day/night, and no additional lighting was supplied.

Nine *B. rapa* accessions (Table 2) with a wide range of flowering times were used to screen for *BrFLC2* sequence variations. The accessions were grown in a greenhouse in Beijing in the fall of both 2008 and 2009 to investigate variations in flowering times.

A BC_2_DH population with 120 lines derived from a cross between the yellow sarson line L143 (R500 from Wisconsin University) and the Chinese cabbage line Z16, using Z16 as the recurrent parent, were screened for polymorphisms of the BrFLC2Indel marker. We selected five lines with the insertion allele and three lines with the deletion allele and grew them in the greenhouse in Beijing from 20 August 2010 to investigate flowering time. Five replicates were grown for each line. Flowering time was scored as the number of days from sowing to the opening of the first flower (DTF). For the nine *B. rapa* accessions that were used to sequence *BrFLC2*, DTF was recorded as described above until 90 d and then those plants with no open flowers were transferred to a cold treatment. For the germplasm collection of 159 accessions grown in the open field, DTF was recorded until 155 DTF, while in the greenhouse flowering time was recorded until 150 DTF. Plants that did not have open flowers at the end of the experiment were assigned a value of 90 DTF, 155 DTF, and 150 DTF in the three respective experiments.

### *BrFLC2* amplification

Genomic DNA was isolated from leaf samples using the CTAB method [28]. Specific primers were designed for *BrFLC2* (AY205317S1, AY205317S2). *BrFLC2* was amplified by nested PCR. The outside forward primer was FLC012F1 (5′- CCTTGATCGATATGGGAAACAAC -3′) located in exon 2 and the outside reverse primer was FLCR5 (5′- TAATTAAGYAGYGGGAGAGTYAC-3′) located in exon 7. The inside forward primer was FLC2F8 (5′-GGAATCAAATTCTGATGTAAGCGTC -3′) located in exon 4 and the inside reverse primer was FLC2R6 (5′-TTTGTCCAGGTGACATCTCCATT-3′) located in exon 7 (Figures 1). The amplified fragment covered the region of exons 4–7 and the intervening introns between these exons. PCR was carried out in a total volume of 20 μl containing 50 ng template DNA, 0.5 μM each primer, 200 μM dNTPs, 1× PCR reaction buffer, and 1 U *Taq* polymerase. PCR was performed under the following conditions: the template was denatured at 94°C for 5 min, followed by 35 cycles of amplification (94°C for 1 min, 56°C for 1 min, 72°C for 1 min 30 s), and a final extension at 72°C for 10 min. PCR products from the nine accessions listed in Table 2 were purified by ethanol and NaAc (3 M, pH = 5.4) and then cloned into the PMD-18 T vectors (Promega, http://www.promega.com) for sequencing.

### InDel marker analysis and CAPS marker analysis

The forward primer FLC2IndelF (5′-GTCGACTCCCTCGTTCAGC -3′) in exon 4 and the reverse primer FLC2IndelR (5′-AGGGAAACTAATACAATACGCAA -3′) in intron 5 were designed to develop an InDel marker for *BrFLC2*. PCR was performed under the following conditions: denaturation at 94°C for 3 min, followed by 35 cycles of amplification (94°C for 45 s, 55°C for 45 s, 72°C for 1 min), and a final extension at 72°C for 10 min. The PCR products were fractionated on an 8.0% polyacrylamide gel to determine the genotype of the InDel marker. We used the CAPS marker for *BrFLC1*, FLC1-*MvaI,* to screen the 159 accessions as described by Yuan *et al*. [18].

### RNA extraction and reverse-transcriptase PCR (RT-PCR)

Germinated seeds from the yellow sarson accession L143 were planted in pots in a growth chamber at 25/20°C (day/night) with a 16-h light/8-h dark photoperiod. Young leaves were collected from plants 14 days after sowing. Total RNA was extracted using TRIplant Reagent (BioTeke, http://www.bioteke.com/). First-strand cDNA was synthesized from 1 μg total RNA using a cDNA synthesis kit (MBI Fermentas, http://www.fermentas.com) according to the manufacturer’s instructions. Because there are four homologs of FLC in *B. rapa*, we designed specific primers to amplify to amplify the cDNA of *BrFLC2*, based on the *B. rapa* genome sequence (http://brassicadb.org/brad/). The specific primers were as follows: forward, BrCFLC2F (5′-CCGAACCTCAGGATCAAATT -3′) located in the 5′UTR, and reverse, BrCFLC2R (5′ -TTCACCCTTATAGGGGAATAGTT -3′) located in the 3′UTR. We carried out RT-PCR under the following conditions: denaturation at 94°C for 4 min, followed by 35 cycles of amplification (94°C for 45 s, 60°C for 45 s, 72°C for 1 min), and a final extension at 72°C for 10 min. The amplified products were separated on SYBR green-stained 2.0% agarose gels. RT-PCR products were precipitated using ethanol and then dissolved in ddH_2_O. The purified RT-PCR product was cloned into pEASY-T1 vectors (TransGen Biotech, http://www.transgen.com) for sequencing. *Escherichia coli* strain DH5a (Tiangen Biotech, http://www.tiangen.com/) was transformed with these constructs and the positive colonies were selected. Plasmid DNA was isolated and sequenced using corresponding primers.

### Sequence analysis

The *BrFLC2* gene was sequenced using an ABI3730XL DNA analyzer (Perkin-Elmer, USA). All sequences were aligned against the published *BrFLC2* sequence. Sequence alignment and analysis were conducted using MegAlign of DNASTAR.

### Statistical analysis

Analysis of variance (ANOVA) and analysis of association were tested by one-way ANOVA and one-tailed Pearson correlation in SPSS version 12.0.1 (SPSS Inc., Chicago, IL, USA). ANOVA was performed with marker genotype as a factor.

## Abbreviations

FLC: flowering locus C; InDel: insertion/deletion; DH: doubled haploid; DTF: days to flowering.

## Authors’ contributions

JW and XW designed experiments. KW, JW, and JZ conducted marker analysis and analyzed data. FC performed sequence analysis. SL and QW conducted field experiments and phenotype investigations. JW, XW, and GB drafted and revised the manuscript. All authors read and approved the final manuscript.

## Supplementary Material

Additional file 1 **Flowering time and genotype of*BrFLC1*and*BrFLC2*in 159 accessions in*B. rapa*germplasm collection.** Click here for file

Additional file 2 **Sequence of fragments with insertion/deletion across exon 4 and intron 4 amplified from nine*B. rapa*accessions.** (DOC 105 kb) Click here for file
