# Supplementary material for: A naturally occurring InDel variation in BraA.FLC.b (BrFLC2) associated with flowering time variation in Brassica rapa
Source: BMC Plant Biol. 2012 Aug 28;12:151. doi: 10.1186/1471-2229-12-151 (PMC3487953; doi:10.1186/1471-2229-12-151)

**Supplementary File 2** The alignment of the amplified fragments to identify the InDel polymorphisms of *BrFLC2*.


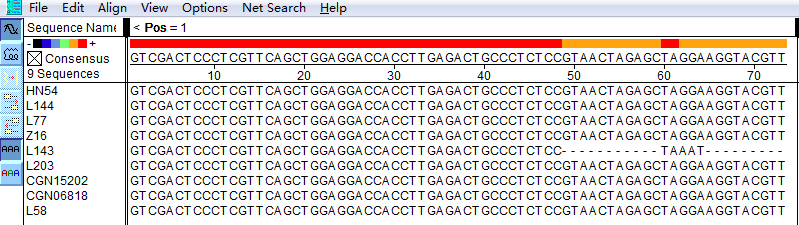


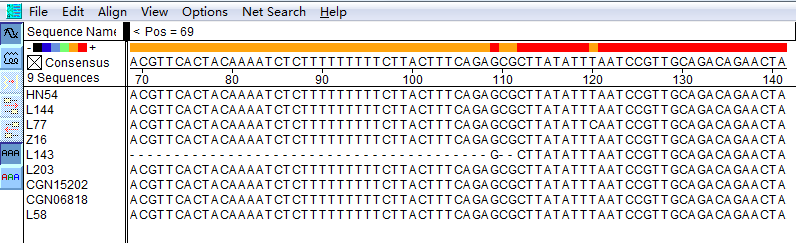


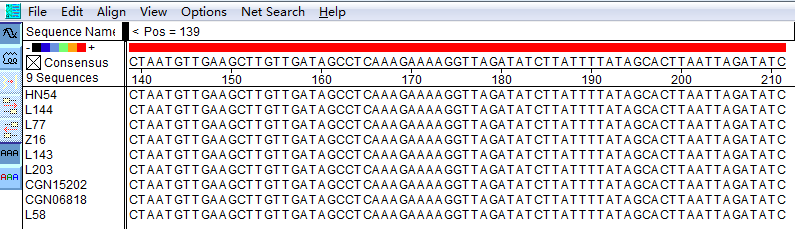


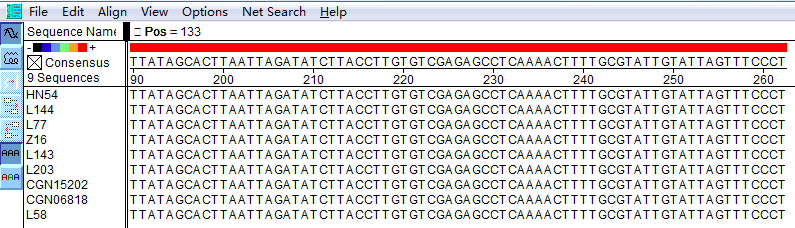

Supplement: Additional file 2 — Sequence of fragments with insertion/deletion across exon 4 and intron 4 amplified from nineB. rapaaccessions. (DOC 105 kb) [file 1471-2229-12-151-S2.doc]
